# Emergency attendance for acute hyper- and hypoglycaemia in the adult diabetic population of the metropolitan area of Milan: quantifying the phenomenon and studying its predictors

**DOI:** 10.1186/s12902-020-0546-1

**Published:** 2020-05-19

**Authors:** Anita Andreano, Marco Bosio, Antonio Giampiero Russo

**Affiliations:** 1 Epidemiology Unit, Agency for Health Protection (ATS) of Milan, C.so Italia, 19, 20122 Milano, Milan, (MI) Italy; 2 General Directorate, Agency for Health Protection (ATS) of Milan, Milan, Italy

**Keywords:** Acute hypoglycaemia, Acute hyperglycaemia, Adult diabetes, Diabetes complications epidemiology

## Abstract

**Background:**

We quantified, among diabetic adults, the frequency, costs, and factors associated with visits to the emergency department (EDs) and subsequent hospitalizations for acute hypoglycaemic and hyperglycaemic events.

**Methods:**

We included adults with diabetes residing in the Milan Agency for Health Protection between 2015 and 2017. From healthcare databases, we identified demographic variables, comorbidities, type of treatment, insulin treatment duration, previous ED attendances for acute glycaemic events, and two indicators of glycaemic monitoring. Using a validated ICD-9-CM coding algorithm, we identified all ED attendances for acute glycaemic events from the ED database and calculated their incidence. We computed the direct costs from health databases and presented them as average annual mean costs for those having had at least an ED attendance. The analysis of the association between the number of ED attendances and potential determinants was performed using zero-inflated negative binomial regression models. These two-part models concomitantly estimate two sets of parameters: the odds-ratios (ORs) of having no attendances and the incidence rate ratios (IRRs) of attendance.

**Results:**

The cohort included 168,285 subjects, 70% of subjects were older than 64 years, 56% were males, and 26% were treated with insulin. The incidence of acute glycaemic events for those attending the ED was 7.0 per 1000 patient-years, followed by hospitalization 26.0% of the time. The total annual direct cost for ED attendances due to acute glycaemic events was 174,000 €. Type of antidiabetic treatment had the strongest association with ED attendances for hypoglycaemia. Patients assuming insulin only had a lower probability of having no attendances (OR compared to those who assumed non-insulin antidiabetic drugs =0.01, 95% CI = 0.00–0.02). These patients also had the highest rate of hyperglycaemic episodes (IRR = 7.7, 95% CI = 5.1–11.7 for insulin only vs. non-insulin antidiabetic drugs). Subjects having had a previous episode of the same type leading to an ED visit had a higher rate of subsequent attendances (IRR for hypoglycaemia = 5.3, 95% CI = 3.9–7.3 and IRR for hyperglycaemia = 3.7, 95% CI = 1.3–10.2).

**Conclusion:**

Insulin treatment and having had a prior acute glycaemic event leading to an ED visit were major predictors of ED attendance for hyper and hypoglycaemia in a population of adults with diabetes.

## Background

Severe acute glycaemic events are a persistent and potentially avoidable cause of attendance to the emergency department (ED) in adult patients with type 1 and 2 diabetes mellitus (DM) [1–4]. These events include acute hyperglycaemic events leading, in their most severe forms, to a hyperosmolar hyperglycaemic state (HHS), diabetic ketoacidosis (DKA), and hypoglycaemic episodes. The latter are frequently caused by the use of glucose-lowering drugs, especially those that increase insulin levels [1].

Quantifying the phenomenon and comparing it across health services is complex because of the various definitions of severe hyper and hypoglycaemia, especially when the analysis is performed at the population level [4]. For severe acute hyperglycaemic events, the incidence is estimated to be in the range of 0.5 to 8 episodes per 1000 person-years in type 2 diabetes [5], and the range is even wider in people with type 1 diabetes, from 0 to 56 per 1000 person-years [6]. The evaluation of trends over time, specifically during the 2006–2011 period, showed that overall ED attendance rates for hyperglycaemia remained stable in the United States, but increased by 17% in 65–74 year-olds and by one third in women. In the same period, hypoglycaemic emergency attendances diminished by 22% in all age groups, excluding young adults [7]. Those episodes have a detrimental effect on the quality of life for individuals with diabetes [8, 9], but also pose an economic burden on the healthcare system [10, 11]. Direct medical costs attributable to acute hypoglycaemia episodes were estimated to be more than 13 million pounds/year in the United Kingdom [12]. In the United States, costs derived from emergency attendance and hospitalization related to the acute loss of glycaemic control in patients with DM were estimated to be nearly 2.8 billion dollars/year [11]. The frequency and costs related to these episodes depend on the organization of integrated diabetic care in a territory [13]. In Italy, the data regarding this phenomenon are scarce, are limited to hypoglycaemia, and are not estimated on a population basis [14–16].

Factors associated with DKA and HHS in adult patients with Type 1 and Type 2 diabetes are inadequate glycaemic control, including the discontinuation of insulin therapy, concomitant acute diseases (more commonly an infection), and the concurrent use of several drugs, including diuretics, glucocorticoids, atypical antipsychotics, and cocaine [17, 18]. Factors associated with episodes of hypoglycaemia in the same population are age, both early adulthood and old age, low socioeconomic status, type of glucose-lowering treatment, a long duration of insulin therapy, prior episodes of severe hypoglycaemia, diabetes complications, particularly autonomic neuropathy and chronic kidney disease, and cognitive impairment [19]. In Type 1 diabetes, genetic factors also play a role in the risk and awareness of hypoglycaemia [20, 21]. Research on the risk factors has mainly been conducted in patients who were referred to diabetes clinics or referral hospitals [6, 22].

However, to prospectively identify the at-risk patient groups who need prevention policies to be strengthened, it is necessary to confirm the role of these factors and study their comparative relevance in the general population, including patients followed by their general practitioner or not under periodical medical surveillance.

We aimed to quantify attendances to EDs for the acute loss of glycaemic control, the costs of these attendances, and to establish the characteristics of patients and treatments associated with a higher number of attendances. Secondarily, we analysed the risk of hospitalization after attendance to the ED for hypo or hyperglycaemia.

## Methods

### Population, data sources, and variable definitions

All subjects with DM residing in the territory of the Milan Agency for Health Protection (AHP) from 1 January 2015 to 31 December 2017 were identified using current healthcare databases and algorithms defined by the Lombardy Region (Regional Decision No. 6164 and No. 7655) (Additional file 1, Table A1) [23]. Subjects who were not registered with the regional universal coverage healthcare system could not be identified. Therefore, temporary residents in the area, who were either registered with another regional system or not registered at all in the NHS, were excluded. Gestational diabetes was also excluded from the algorithm. We included patients with a DM diagnosis, both type 1 and type 2. We excluded subjects below the age of 18 and patients who were not pharmacologically treated. Patients were followed-up for acute glycaemic events until 31 August 2018 through the ED database of the AHP, including the healthcare consumption of resident patients outside the area. Data anonymization was provided by an internal code used in every administrative database and was used for deterministic record linkage on a unique identifier.

### Individual characteristics and treatment characteristics

Demographic variables, including age and gender, were extracted from the current healthcare databases of the AHP. The socioeconomic deprivation index quintile of the census section, calculated from data provided by the National Institute of Statistics (ISTAT) and normalized to the AHP, was assigned based on the address of residence [24]. The anti-diabetic treatment was determined using the drug prescription database and categorized as: insulin alone, insulin and non-insulin anti-DM drugs, non-insulin anti-DM drugs including at least one at risk for hypoglycaemia, and other non-insulin anti-DM drugs only. ATC codes included in each category are reported in Additional file 1, Table A3. From the current healthcare databases, we also determined the total number of comorbidities and the presence of chronic renal failure (CKD) and peripheral neuropathy (PN). The databases, algorithms, and codes used to define comorbidities are described in Additional file 1, Table A3. From the drug prescription database, we calculated the duration of insulin treatment, dichotomized as less vs. equal or greater than 5 years (for the algorithm, see Additional file 1, Table A3). For each individual, we also calculated two indicators of adequate glycaemic monitoring: undergoing or not undergoing glycated haemoglobin (HbA1c) level testing at least once a year, and receiving a number of glucose test strips every year from the regional healthcare service considered as adequate for the type of treatment, according to national guidelines (algorithms described in Additional file 1, Table A3).

### Definition of the outcome acute glycaemic event

Attendances to the ED for acute glycaemic events were identified for the cohort, through deterministic record linkage with the ED database of the AHP. We selected ED records having ICD-CM codes of acute hypo or hyperglycaemia in the diagnosis field; version 9, which is the version currently adopted in Italy, was used. To determine the codes, a validated algorithm to identify hypoglycaemia from claims was reviewed [25], and the codes were abstracted (Additional file 1, Table A2). No validated algorithm for hyperglycaemia was found, so potential codes were individuated from the ICD-9-CM codes list (Additional file 1, Table A2). Also, as it is the use of coding partially specific to a healthcare system, we validated the use of the identified ICD-9-CM codes in the ED database of the AHP, by means of reviewing the full clinical records of hospital admission following ED attendance in a random sub-sample of the cohort, as described in Additional file 1, additional methods. The codes selected after this validation were used to identify acute glycaemic events, overall and distinguished, in hypo and hyperglycaemia, for the study cohort. After identifying the events, we determined which attendances to the emergency department were followed by a hospital admission, using a specific variable present in the ED dataset.

### Primary and secondary endpoints

#### Estimation of incidence, prevalence, and costs of ED attendances for acute glycaemic events

We calculated the incidence and prevalence of the glycaemic events, overall and separately, for hyper and hypoglycaemia, and hospitalization following attendance to the ED. Furthermore, we estimated the total annual direct costs for the regional health system for each subject considering hospitalizations, attendances to the ED, outpatient visits and diagnostic tests, drugs, glucose test strips, and other glucose monitoring devices. For each health event, the corresponding tariff in the administrative database was used. Moreover, medical direct costs were estimated separately for all acute glycaemic, hypoglycaemia, and hyperglycaemia ED attendances, summing the total amount for each attendance as reported in the ED database.

For records with no reported total amount, the average cost for each ICD-9-CM diagnosis code was imputed. The costs were calculated for each patient per year, and the average annual cost for the different categories of subjects was calculated for the 2015–2017 period.

#### Analysis of the association between ED attendances for hypoglycaemia and patient characteristics

We analysed the association between hypoglycaemia and a priori selected demographic and treatment characteristics, potentially associated with hypoglycaemia risk from the literature [3, 5, 6, 17, 19, 22, 26] and available through the current healthcare databases. The patient characteristics were gender, age class, deprivation index of the census section, having a diagnosis of CKD, presence of PN, type of anti-diabetic treatment as defined in the previous paragraph, having received an adequate number of glucose test strips, having had at least one ED attendance for hypoglycaemia in 2014, and duration of insulin treatment. We performed the analysis using a multivariable regression two-part model to account for the considerable number of patients having no event. This model assumes that there is a certain individual susceptibility to hypoglycaemia (i.e. a non-negligible probability of ED attendance for hypoglycaemia) and that it depends on significant factors in the logistic regression model. The second part of the model assumes that the rates of ED attendance for hypoglycaemia of susceptible individuals depend on factors identified through a negative binomial model, accounting for extra-variability. The role of individual factors on the probability of not being susceptible is reported by an odds ratio (OR), while their effect on the rate of events in susceptible individuals are reported by an incidence rate ratio (IRR).

The same explanatory variables were included in the multivariable Poisson regression model which evaluated the risk of hospitalization after an ED for acute hypoglycaemia.

#### Analysis of the association between ED attendances for hyperglycaemia and patient characteristics

In the acute hyperglycaemia analysis on the whole cohort, we excluded all ED attendances leading to the first diagnosis of DM as explained in the Additional methods (Additional file 1). With the same approach described for hypoglycaemia, in the analysis of factors associated with ED attendances for hyperglycaemia, we included the following characteristics in the models [17, 18]: gender, age class, deprivation index, hypoglycaemia treatment, glycated haemoglobin test, and ED attendance for a hyperglycaemic event in 2014. A zero-inflated negative binomial multivariable regression model was used for the same reasons described for hypoglycaemia.

Moreover, the same explanatory variables were explored in the Poisson multivariable regression model which evaluated the risk of hospitalization after an ED attendance for acute hyperglycaemia.

### Statistical analysis

The characteristics of the cohort were described using absolute numbers and percentages, overall, and stratifying over ED attendances for an acute glycaemic event, hypoglycaemia, and hyperglycaemia (excluding those leading to DM diagnosis). Groups were compared using the χ^2^ test. Costs were described using mean, median, standard deviation (s.d.), and quartiles (Q).

Factors associated with the frequency of ED attendances for hypoglycaemia were first assessed using a Poisson model. Due to overdispersion and the excess of patients not experiencing the event with respect to a Poisson distribution, negative binomial (NB), zero-inflated Poisson (ZIP), and zero-inflated negative binomial (ZINB) regression models were also fitted [27, 28]. To select the best fitting model, we used the Vuong test [29]. To allow for the skewed distribution of the duration of the follow-up, we included in each model, an offset variable (natural logarithm of the individual total follow-up time) [28, 30]. Demographic and treatment variables potentially associated with hypoglycaemia risk from the literature, and available through the current healthcare databases, were included in all the multivariable regression models [3, 5, 6, 17, 19, 22, 26]. The same variables were included also in the ZIP and ZINB inflate component of the models, which predict whether a patient would be an excess zero. The same approach was adopted to evaluate the potential factors associated with ED attendances for hyperglycaemia, excluding those related to the diagnosis. The results of the models are presented as an OR of being an excess zero (i.e. not being susceptible to the event) for the inflate portion, and an IRR of the event for the count part, along with their corresponding 95% confidence intervals (CI). We also evaluated the association between demographic and treatment characteristics and the incidence of hospitalization after an ED attendance in patients having at least one acute hypoglycaemic event, and in those who experienced at least one hyperglycaemic event. In the absence of overdispersion and excess of non-events, a multivariable Poisson model was adopted for both, and the results were presented as IRRs along with their corresponding 95% CI. All analyses were performed using SAS software (v 9.4, SAS Institute Inc., Cary, NC, USA).

## Results

### Acute glycaemic event identification

The ED attendances for acute glycaemic events identified with the provisional ICD-9-CM codes (Additional file 1, Table A2) were *n* = 2510; five of the initially selected codes were never found in the ED database (250.81, 250.83, 250.90, 250.91, 251.1). The hospital stratified random sample of ED attendances, followed by hospitalization, included 291 records. The full clinical chart, including the ED report, was available and reviewed for 261 cases (89.7% of sampled records). The characteristics of the sample and its comparability with the whole cohort are presented in Additional file 1, Table A4. From the evaluation of the clinical records, 10 hospitalizations were performed for reasons other than an acute glycaemic alteration (False discovery rate FDR = 3.8%). Of the remaining 251 episodes, 117 were hypoglycaemic (46.6%) and 134 were hyperglycaemic (53.4%). Of the latter, 27 (10.3% of examined records) were hospitalizations following the first diagnosis of diabetes during the ED attendance, and in these records, the 80th percentile of the distribution of the time interval between the date of diagnosis in the database of chronic disease and the date of the ED in the sample was 15 days. Among patients with hypoglycaemia, 2.8% were treated with glucagon and 91.6% with glucose and/or glucose solution i.v. in the emergency room. Among patients with hyperglycaemia, insulin was administered in the emergency room in 83.7% of cases. Three-point-5 % of patients with hypoglycaemia and 10.4% of those with hyperglycaemia, were admitted to an intensive care unit. Six-point-1 % of hospitalizations for hypoglycaemia and 4.8% for hyperglycaemia, led to the death of the patient.

All provisional codes had a FDR for acute glycaemic events lower than 30% (Additional file 1, Table A2); consequently, they were all retained in the final algorithm to identify ED attendances for acute glycaemic events. Three codes had a Positive Predictive Value (PPV) lower than 80%: “250.82 Diabetes with other specified manifestations, type II or unspecified type, uncontrolled”, PPV for hyperglycaemia 60%; “276.2 Acidosis”, PPV for hyperglycaemia 56%, and “790.29 Other abnormal glucose” PPV for hyperglycaemia 75. These codes had identified 38 records in the sample (14.6% of the reviewed clinical records). They were not included in the final algorithms used to identify distinctively hyper or hypoglycaemia events in the cohort (Additional file 1, Table A2).

### Incidence and prevalence of acute glycaemic events in the cohort

**Fig. 1:**
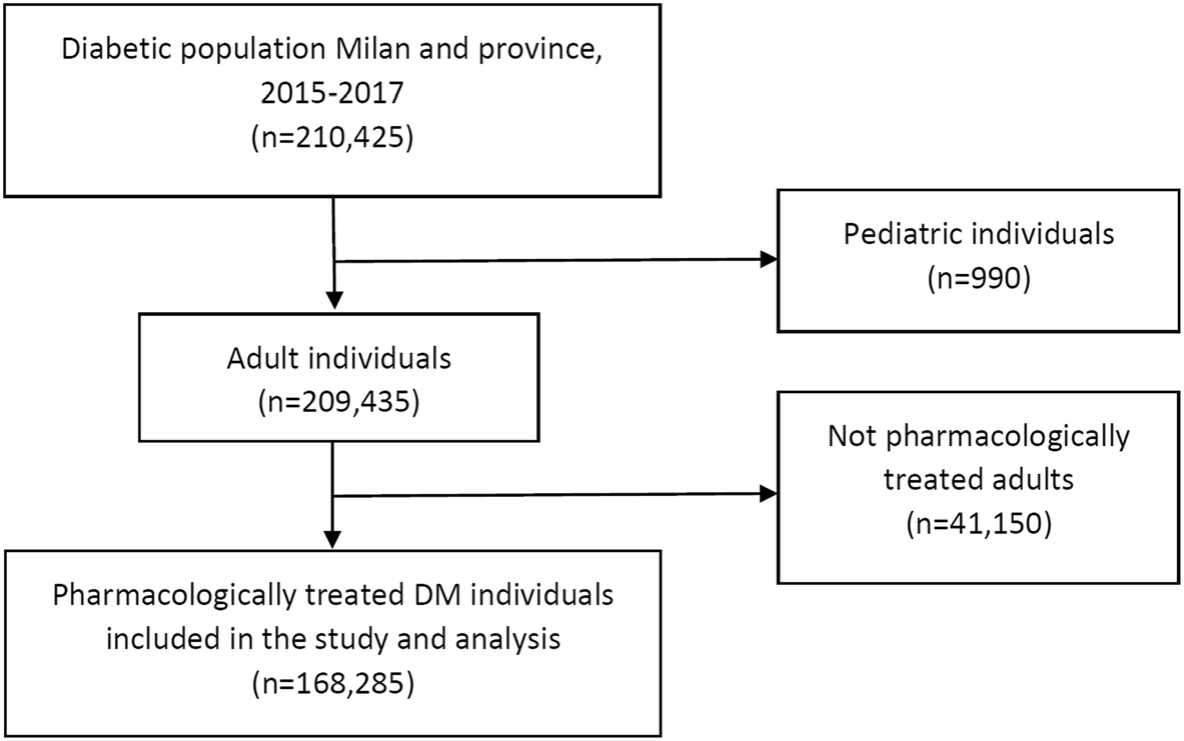
Selection of the cohort

A total of 210,425 persons with DM resided in the Milan Metropolitan area between 2015 and 2017. Age-standardized (reference population ESP 2013 [31]) prevalence of DM at 31 December 2017 was 4.9% (5.7% in men and 4.1% in women). In 2017, the age-standardized incidence of DM was 36.6 for 10,000 residents (38.3 in men and 34.9 in women). For insulin-dependent diabetes (IDD), the incidence was 4.9 for 10,000 in both genders. Excluding paediatric subjects (*n* = 990) and untreated adult patients (*n* = 41,150), the total number of subjects included in the analysis was 168,285 (Fig. 1). Almost seventy percent of the cohort was older than 64 years, 56% were male and half resided in a census section included in the two most deprived quintiles (Table 1). Nearly 6% of the cohort had CKD, and 16% had PN. Slightly over a quarter of individuals used insulin, and 43% for at least 5 years. Table 2 describes the characteristics of the subject experiencing at least one attendance to the emergency department for hypoglycemia and for hyperglycemia.

**Table 1:** Characteristics of the diabetic adult population residing in the Health Agency of Milan, 2015–17

Characteristic	Total *(N = 168,285)*	One or more attendance to the ED for acute glycemic events			***P*** value^b^
		No *(N = 165,121)*	Yes *(N = 3164)*		
	Number (%)	Number (%)	Number (%)	% of total	
Gender					< 0.001
Female	73,715 (43.8)	72,182 (43.7)	1533 (48.5)	2.1	
Male	94,570 (56.2)	92,939 (56.3)	1631 (51.5)	1.7	
Age class (years)					< 0.001
≤ 44	8683 (5.2)	8407 (5.1)	276 (8.7)	3.2	
45–54	16,298 (9.7)	16,025 (9.7)	273 (8.6)	1.7	
55–64	32,210 (19.1)	31,827 (19.3)	383 (12.1)	1.2	
65–74	51,056 (30.3)	50,412 (30.5)	644 (20.4)	1.3	
≥ 75	60,038 (35.7)	58,450 (35.4)	1588 (50.2)	2.6	
Deprivation index					< 0.001
I-II	39,307 (23.4)	38,593 (23.4)	714 (22.6)	1.8	
III	44,453 (26.4)	43,695 (26.5)	758 (24.0)	1.7	
IV-V	84,525 (50.2)	82,833 (50.2)	1692 (53.5)	2.0	
Number of comorbidities					< 0.001
None	22,181 (13.2)	21,839 (13.2)	342 (10.8)	1.5	
One	37,599 (22.3)	37,178 (22.5)	421 (13.3)	1.1	
Two or more	108,505 (64.5)	106,104 (64.3)	2401 (75.9)	2.2	
Chronic renal failure					< 0.001
No	158,661 (94.3)	155,964 (94.5)	2697 (85.2)	1.7	
Yes	9624 (5.7)	9157 (5.5)	467 (14.8)	4.9	
Peripheral neuropathy					< 0.001
No	141,424 (84.0)	138,991 (84.2)	2433 (76.9)	1.7	
Yes	26,861 (16.0)	26,130 (15.8)	731 (23.1)	2.7	
Hypoglycemia treatment					< 0.001
Insulin alone	21,817 (13.0)	20,589 (12.5)	1228 (38.8)	5.6	
Insulin and non-insulin anti-DM drugs	22,185 (13.2)	21,269 (12.9)	916 (29.0)	4.1	
Anti-DM drugs at risk of hypoglycemia^a^	51,838 (30.8)	51,115 (31.0)	723 (22.9)	1.4	
Other non-insulin anti-DM drugs only	72,445 (43.0)	72,148 (43.7)	297 (9.4)	0.4	
Duration of insulin treatment					< 0.001
Less than 5 years	24,891 (14.8)	23,910 (14.5)	981 (31.0)	3.9	
5 years or more	19,111 (11.4)	17,948 (10.9)	1163 (36.8)	6.1	
No insulin treatment	124,283 (73.9)	123,263 (74.7)	1020 (32.2)	0.8	
ED attendance for acute glycemic event in 2014					< 0.001
No	167,528 (99.6)	164,484 (99.6)	3044 (96.2)	1.8	
Yes	757 (0.4)	637 (0.4)	120 (3.8)	15.9	
Glycated hemoglobin test					0.024
No	67,101 (39.9)	65,778 (39.8)	1323 (41.8)	2.0	
Yes	101,184 (60.1)	99,343 (60.2)	1841 (58.2)	1.8	
Adequate number of glucose test strips					< 0.001
No	96,420 (57.3)	94,800 (57.4)	1620 (51.2)	1.7	
Yes	71,865 (42.7)	70,321 (42.6)	1544 (48.8)	2.1	

^a^excluded insulin; ^b^ χ^2^ test**;** DM, diabetes mellitus; ED emergency department

**Table 2:** Characteristics of the subjects experiencing at least one attendance to the emergency department for hypoglycemia and for hyperglycemia

Characteristic	Hypoglycemia N of pts. = 2137			Hyperglycemia^a^ N of pts. = 633		
	N (%)	% of total	***P*** value^b^	N (%)	% of total	***P*** value^b^
Gender			< 0.001			< 0.001
Female	1018 (47.6)	1.4		332 (52.4)	0.5	
Male	1119 (52.4)	1.2		301 (47.6)	0.3	
Age class (years)			< 0.001			< 0.001
≤ 44 years	148 (6.9)	1.7		89 (14.1)	1.0	
45–54	147 (6.9)	0.9		77 (12.2)	0.5	
55–64	214 (10.0)	0.7		95 (15.0)	0.3	
65–74	427 (20.0)	0.8		125 (19.7)	0.2	
≥ 75	1201 (56.2)	2.0		247 (39.0)	0.4	
Deprivation index			0.014			0.035
I-II	472 (22.1)	1.2		140 (22.1)	0.4	
III	525 (24.6)	1.2		144 (22.7)	0.3	
IV-V	1140 (53.3)	1.3		349 (55.1)	0.4	
Number of comorbidities			< 0.001			< 0.001
None	195 (9.1)	0.9		87 (13.7)	0.4	
One	250 (11.7)	0.7		92 (14.5)	0.2	
Two or more	1692 (79.2)	1.6		454 (71.7)	0.4	
Chronic renal failure			< 0.001			< 0.001
No	1814 (84.9)	1.1		546 (86.3)	0.3	
Yes	323 (15.1)	3.4		87 (13.7)	0.9	
Peripheral neuropathy			< 0.001			< 0.001
No	1651 (77.3)	1.2		459 (72.5)	0.3	
Yes	486 (22.7)	1.8		174 (27.5)	0.6	
Hypoglycemia treatment			< 0.001			< 0.001
Insulin alone	891 (41.7)	4.1		232 (36.7)	1.1	
Insulin and non-insulin anti-DM drugs	607 (28.4)	2.7		211 (33.3)	1.0	
Anti-DM drugs at risk of hypoglycemia	523 (24.5)	1.0		116 (18.3)	0.2	
Other non-insulin anti-DM drugs only	116 (5.4)	0.2		74 (11.7)	0.1	
Duration of insulin treatment			< 0.001			< 0.001
Less than 5 years	572 (26.8)	2.3		239 (37.8)	1.0	
5 years or more	926 (43.3)	4.8		204 (32.2)	1.1	
No insulin treatment	639 (29.9)	0.5		190 (30.0)	0.2	
ED attendance for hypoglycemia in 2014			< 0.001			
No	2056 (96.2)	1.2				
Yes	81 (3.8)	17.3				
ED attendance for hyperglycemia in 2014						< 0.001
No				617 (97.5)	0.4	
Yes				16 (2.5)	6.6	
Glycated hemoglobin test			0.65			< 0.001
No	842 (39.4)	1.3		303 (47.9)	0.5	
Yes	1295 (60.6)	1.3		330 (52.1)	0.3	
Adequate number of glucose test strips			< 0.001			0.79
No	1026 (48.0)	1.1		366 (57.8)	0.4	
Yes	1111 (52.0)	1.5		267 (42.2)	0.4	

^a^excluding events leading to DM diagnosis**;**^b^ χ^2^ test**;** DM, diabetes mellitus; ED, emergency department

The total number of ED attendances of the cohort for any cause between 1st January 2015 and 31st August 2018 was 270,594 (mean number of events per subject: 1.61, sd: 2.64) in 99,174 subjects (58.9% of the cohort), with an incidence of 0.5 per patient-year. One-point-9 % of the cohort (*n* = 3164 subjects) experienced 3719 acute glycaemic events leading to an ED attendance (mean number of events per subject: 0.02, sd: 0.18), with an incidence of 7.0 per 1000 patient-years. Of those, 967 attendances were followed by hospitalization (26.0%). Excluding ED attendances within 15 days from DM diagnosis, i.e. presumably related to diagnosis (*n* = 263, 7.1%), the incidence was 6.5 per 1000 patient-years. The validated coding algorithms revealed that, for 348 events, a definitive characterization as hyper or hypoglycaemia was not possible (9.3% of all acute glycaemic events). Excluding not characterized and related to diagnosis ED attendances, 48 patients (1.7%) experienced hyper and hypoglycaemia events during follow-up. Those subjects were included in both analyses.

### Costs of acute glycaemic events in the cohort

In the period between 2015 and 2018, the total patient annual cost of ED attendances for all acute glycaemic events was 174,544 euros, with a median cost per attendance of 137 euro (Q1, Q3 = 87–233 euros). The total patient annual cost for acute hypoglycaemia was 108,648 euros, with a median cost per attendance of 127 euros (Q1, Q3 = 83–227 euro). For acute hyperglycaemia, the cost was 44,697 euros with a median cost per attendance of 142 euros (Q1, Q3 = 92–226 euros). No relevant differences were detected between subjects treated with insulin and those who were not (data not shown). The median total direct annual costs for patients having at least one ED attendance between 2015 and 2017 were 2.3 times higher than those of patients not experiencing any ED attendances (median = 2533 and Q1, Q4 = 1140–5931 vs. median = 5839 and Q1, Q3 = 3052–11,757 euro, Table 3). Costs were higher, both for subjects without and with ED attendances, among elderly patients, if comorbidities were present and if they were treated with insulin. Patients with these characteristics were more represented among patients with ED attendances (Table 1), particularly 68% of patients experiencing ED attendances for an acute glycaemic event were treated with insulin vs. 25% among those who were not.

**Table 3:** Annual total direct costs for subjects with diabetes having and not an attendance to the emergency department for acute glycemic events

Emergency department attendances	Characteristic	N of subjects	Average annual direct costs (euro)				
			Mean	Std Dev	Median	Lower Quartile	Upper Quartile
None		*162,965*	5584	9550	2533	1140	5931
At least one for any glycemic event		*3121*	10,055	12,603	5839	3052	11,757
At least one for any glycemic event followed by hospitalization		*897*	11,474	12,413	7555	3818	13,872
**Age class (years)**							
None	≤ 44	*8381*	3459	8786	1934	685	3383
	45–54	*15,973*	4306	10,275	1407	569	3456
	55–64	*31,666*	4843	10,234	1818	812	4383
	65–74	*49,991*	5876	9591	2659	1271	6239
	≥75	*56,954*	6411	8879	3433	1642	7662
At least one for any glycemic event	≤ 44	*275*	5879	8111	3438	2474	5909
	45–54	*273*	8260	15,231	3538	1844	7588
	55–64	*383*	12,711	17,260	5828	2787	14,708
	65–74	*641*	12,764	14,651	7797	4204	16,492
	≥75	*1549*	9336	9824	6429	3426	11,652
At least one for any glycemic event followed by hospitalization	≤ 44	*86*	8083	11,505	3979	2855	8109
	45–54	*66*	10,678	17,694	4972	2625	9251
	55–64	*108*	13,971	13,658	9197	3458	19,893
	65–74	*168*	15,289	15,629	9382	5213	20,158
	≥75	*469*	10,266	9346	7540	4247	13,012
**N of comorbidities**							
None	None	*21,701*	1655	2751	873	359	2072
	One	*36,824*	2493	4788	1345	708	2620
	Two or more	*104,440*	7490	11,065	3823	1872	8447
At least one for any glycemic event	None	*341*	3436	4982	2634	1478	3831
	One	*414*	4546	5471	3160	1713	5210
	Two or more	*2366*	11,974	13,618	7626	4187	14,333
At least one for any glycemic event followed by hospitalization	None	*79*	5544	9399	3071	2464	5909
	One	*114*	6200	6139	4538	2442	8151
	Two or more	*704*	12,993	13,029	8628	4848	16,720
**Insuline treatment**							
None	Yes	*40,933*	9881	13,676	5107	2834	10,967
	No	*122,032*	4143	7126	1914	915	4331
At least one for any glycemic event	Yes	*2117*	11,319	13,522	6861	3831	13,181
	No	*1004*	7391	9893	3945	1823	9221
At least one for any glycemic event followed by hospitalization	Yes	*552*	12,946	13,388	8608	4709	15,852
	No	*345*	9119	10,257	6000	2707	11,731

### Risk factors for hypoglycaemic events and subsequent hospitalization

There were 2510 Hypoglycaemic events (ICD-9-CM codes: 251.0, 251.2, 962.3) leading to ED attendance, experienced by 2137 patients, with an incidence of 4.7 per 1000 patient-years. The strongest positive univariate associations between hypoglycaemia and patient characteristics were found for (Table 4): 1) having had an ED attendance for hypoglycaemia in 2014 (17.3% of subjects experiencing hypoglycaemia vs. 1.2% among subjects without ED attendances); 2) being treated with insulin (4.1% among those treated with insulin alone and 2.7% among those treated also with other anti-DM drugs vs. 0.2% for non-insulin anti-DM not at risk for hypoglycaemia); 3) having chronic renal failure (3.4% vs. 1.1% among subjects without CKD). For the hypoglycaemic events, the best fitting multivariable regression model was the zero-inflated negative binomial. Younger patients had a higher probability of not being susceptible to ED attendances for hypoglycaemia (OR = 2.21, 95% CI = 1.14–4.27 for ≤44 year-olds and OR = 2.26, 95% CI = 1.24–4.13 for 45–54 year-olds), but if susceptible, had a higher frequency of events than 55–64 year-olds (IRR = 1.93, 95% CI = 1.21–3.08 and 2.21, 95% CI = 1.41–3.46). The opposite was true for older patients (Table 4). The presence of CKD or peripheral neuropathy reduced the probability of not being susceptible to ED attendances for hypoglycaemia (OR = 0.37, 95% CI = 0.22–0.63 for CKD and OR = 0.67, 95% CI = 0.47–0.96 for PN) but did not influence the IRR. Compared to being treated only with non-insulin anti-DM drugs not at risk for hypoglycaemia, all other treatment modalities were strongly associated with a lower probability of not being susceptible to ED attendances for hypoglycaemia (OR = 0.01, 95% CI = 0.00–0.02 for insulin alone to OR = 0.09, 95% CI = 0.04–0.19 for other anti-DM drugs at risk for hypoglycaemia); while, no statistically significant effect was seen for event rate. Having had an ED attendance for hypoglycaemia in 2014 was associated both with a reduced probability of not being susceptible to ED attendances during the study period (OR = 0.20, 95% CI = 0.07–0.53) and an increased event frequency (IRR = 5.34, 95% CI = 3.93–7.26). Subjects treated with insulin for less than 5 years had an increased probability of not being susceptible to ED attendances for hypoglycaemia (OR = 1.82, 95% CI = 1.25–2.65) and a reduced event frequency (IRR = 0.62, 95% CI = 0.52–0.74), compared to those treated for 5 years or more.

**Table 4:** Characteristics associated with attendance to the emergency department for acute hypoglycemia, and with hospitalization after the attendance, in patients with diabetes

Characteristic		Model for risk of ED attendance for acute hypoglycemia^a^								Model for risk of hospitalization after an ED attendance^b^			
		Negative binomial component				Zero inflate component							
		IRR	95%CI		*P* value	OR	95%CI		*P* value	IRR	95%CI		*P* value
Gender	Female vs. male	0.96	0.82	1.11	0.561	0.81	0.61	1.06	0.121	0.98	0.80	1.19	0.811
Age class (years)	ref., 55–64												
	≤ 44 years	1.93	1.21	3.08	0.006	2.21	1.14	4.27	0.018	0.65	0.30	1.40	0.272
	45–54	2.21	1.41	3.46	0.001	2.26	1.24	4.13	0.008	0.77	0.38	1.53	0.448
	65–74	0.70	0.49	1.01	0.054	0.43	0.25	0.74	0.002	1.51	0.96	2.37	0.075
	≥ 75	0.77	0.55	1.09	0.145	0.10	0.06	0.16	< 0.001	1.66	1.10	2.52	0.017
Deprivation index	ref., I-II												
	III	1.01	0.82	1.25	0.899	0.93	0.64	1.35	0.695	0.98	0.73	1.31	0.867
	IV-V	1.11	0.93	1.34	0.241	0.86	0.62	1.20	0.389	1.04	0.81	1.34	0.734
Chronic renal failure	Yes vs. no	1.11	0.92	1.33	0.268	0.37	0.22	0.63	< 0.001	1.42	1.10	1.84	0.008
Peripheral neuropathy	Yes vs. no	0.91	0.77	1.08	0.288	0.67	0.47	0.96	0.030	0.86	0.67	1.11	0.249
Hypoglycemia treatment	ref., Other non-insulin anti-DM drugs only												
	Insulin alone	1.44	0.76	2.74	0.264	0.01	0.00	0.02	< 0.001	0.45	0.30	0.68	< 0.001
	Insulin and non-insulin anti-DM drugs	1.60	0.84	3.05	0.154	0.02	0.01	0.04	< 0.001	0.58	0.38	0.90	0.014
	Anti-DM drugs at risk of hypoglycemia	0.71	0.37	1.36	0.305	0.09	0.04	0.19	< 0.001	0.86	0.59	1.27	0.460
Adequate number of glucose test strips	No vs. yes	1.05	0.90	1.23	0.529	1.30	0.98	1.74	0.071	1.28	1.03	1.59	0.023
ED attenance for hypoglycemia in 2014	Yes vs. no	5.34	3.93	7.26	< 0.001	0.20	0.07	0.53	0.001	1.00	0.55	1.84	0.990
Duration of insulin treatment	Less than vs. equal or more than 5 years	0.62	0.52	0.74	< 0.001	1.82	1.25	2.65	0.002	1.24	0.93	1.64	0.143

^**a**^zero-inflated negative binomial multivariable regression model; ^b^Poisson multivariable regression model**;***IRR* Incidence rate ratio, *OR* Odds ratio, *CI* Confidence interval

The percentage of hospitalization after an ED attendance for hypoglycaemia was 15.3%. The results of the multivariable Poisson model, which evaluated the risk of hospitalization after an ED attendance for hypoglycaemia, are also reported in Table 4. The factors that increased the incidence of hospitalization after an ED attendance for acute hypoglycaemia were: having CKD (IRR = 1.42, 95% CI 1.10–1.84) and not having received an adequate number of glucose test strips (IR = 1.28, 95% CI 1.03–1.59). In contrast, being treated with insulin reduced the risk of hospitalization by more than 50% compared to being treated only with non-insulin anti-DM drugs (IRR = 0.45, 95% CI 0.30–0.68 for insulin alone).

### Risk factors for hyperglycaemic events and subsequent hospitalization

There were 861 Hyperglycaemic events (ICD-9-CM codes: 250.1, 250.2, 250.3, 250.80, 250.92, 250.93, 276.0, 276.4, 790.21) leading to ED attendances, experienced by *n* = 788 patients, with an incidence of 1.63 per 1000 patient-years. Excluding patients close to diagnosis, the events were *n* = 689, experienced by *n* = 633 patients, with an incidence of 1.30 per 1000 patient-years.

The strongest positive univariate associations between hyperglycaemia and patient characteristics were found for (Table 5): 1) having had an ED attendance for hyperglycaemia in 2014 (6.6% of subjects in the cohort experienced hyperglycaemia among those with vs. 0.4% among subjects without an ED attendance in 2014 for hyperglycaemia); 2) being treated with insulin (1.1% among those treated with insulin alone vs. 0.1% for non-insulin anti-DM not at risk for hypoglycaemia); 3) having chronic renal failure: 0.9% vs. 0.3% among subjects without CKD. For the hyperglycaemic acute events, the best fitting multivariable regression model was the zero-inflated negative binomial (Table 5). Patients older than 56 years had a lower probability of not being susceptible to ED attendances for hyperglycaemia (OR = 0.22, 95% CI = 0.08–0.57), but if susceptible, had a lower frequency of events than 45–64-year-old patients (IRR = 0.24, 95% CI = 0.12–0.52). The treatment modality did not have a statistically significant effect on the probability of being susceptible to hyperglycaemia. At the same time, the use of insulin, alone or with other anti-DM drugs, increased the frequency of events compared to being treated only with non-insulin anti-DM drugs not at risk for hypoglycaemia (IRR = 7.72, 95% CI = 5.11–11.66 for insulin alone and IRR = 8.01, 95% CI = 5.21–12.32 for insulin with other anti-DM drugs). Failure to perform a glycated haemoglobin test every year reduced the probability of not being susceptible to ED attendances for hyperglycaemia (OR = 0.65, 95% CI 0.42–0.99) and increased the event rate (IRR = 1.36, 95% CI 1.07–1.72), compared to individuals having performed it. Furthermore, having had an ED attendance for hyperglycaemia in 2014 increased the event frequency (IRR = 3.68, 95% CI = 1.33–10.23). The percentage of hospitalization after an ED attendance for hypoglycaemia was 34.8%. The results of the multivariable Poisson model, which evaluated the risk of hospitalization after an ED attendance for hyperglycaemia, are also reported in Table 5. The factors that increased the incidence of hospitalization after an ED attendance for acute hyperglycaemia were being treated with insulin, doubling the risk (IR = 2.00, 95% CI 1.31–2.05), and not having performed a yearly HbA1c test (IR = 1.37, 95% CI 1.10–1.72).

**Table 5:** Characteristics associated with attendance to the emergency department for acute hyperglycemia, and with hospitalization after the attendance, in patients with diabetes

Characteristic		Model for risk of ED attendance for acute hypoglycemia^a^								Model for risk of hospitalization after an ED attendance^b^			
		Negative binomial component				Zero inflate component							
		IRR	95%CI		*P* value	OR	95%CI		*P* value	IRR	95%CI		*P* value
Gender	Female vs. male	1.51	1.18	1.93	0.001	1.16	0.75	1.80	0.500	1.06	0.85	1.33	0.589
Age class (years)	ref., 45–64												
	≤ 44	0.86	0.40	1.84	0.693	0.54	0.24	1.22	0.139	0.98	0.69	1.39	0.902
	≥ 65	0.24	0.12	0.52	< 0.001	0.22	0.08	0.57	0.002	1.02	0.79	1.32	0.893
Deprivation index	ref., I-II												
	III	0.80	0.58	1.12	0.195	0.70	0.38	1.28	0.247	1.03	0.76	1.41	0.849
	IV-V	0.91	0.68	1.21	0.504	0.64	0.38	1.07	0.092	0.86	0.65	1.12	0.260
Hypoglycemia treatment	ref., Other non-insulin anti-DM drugs only												
	Insulin alone	7.72	5.11	11.66	< 0.001	0.56	0.28	1.12	0.099	2.00	1.31	3.05	0.001
	Insulin and non-insulin anti-DM drugs	8.01	5.21	12.32	< 0.001	0.58	0.28	1.22	0.150	1.36	0.88	2.11	0.170
	Anti-DM drugs at risk of hypoglycemia	1.88	1.22	2.89	0.004	0.78	0.36	1.67	0.521	1.16	0.72	1.87	0.552
Glycated hemoglobin test	No vs. yes	1.36	1.07	1.72	0.012	0.65	0.42	0.99	0.044	1.37	1.10	1.72	0.006
ED attendance for hyperglycemia in 2014	Yes vs. no	3.68	1.33	10.23	0.012	0.05	0.00	61.65	0.413	1.56	0.93	2.63	0.094

^**a**^zero-inflated negative binomial multivariable regression model; ^b^Poisson multivariable regression model; *IRR* Incidence rate ratio, *OR* Odds ratio, *CI* Confidence interval

## Discussion

Attendances to the ED due to a lack of glycaemic control in the DM adult population of the Milan AHP were close to 900/year, with an incidence rate of 7.0 per 1000 patient-years. When looking specifically at hypoglycaemic events, the incidence was 4.7 per 1000 patient-years and 1.3 per 1000 patient-years for severe acute hyperglycaemias, while the exact nature (hyper or hypoglycaemia) of the remaining attendances could not be determined. Results are difficult to compare with the existing literature because of the relatively low prevalence of diabetes in the study area (about 5%) and due to differences in the ascertainment modalities and hypoglycaemia definitions [19, 32, 33]. Population-based studies performed in different countries estimated the incidence of severe hypoglycaemic episodes requiring medical assistance to be around 4 per 1000 person-year for Type 2 diabetes or adult diabetic populations [4, 12, 22, 34–36], which is similar to what we found.

The mean annual costs for patients attending the ED are almost double compared to patients who have never attended. This is attributable to the higher prevalence of patients older than 75 years, with two or more comorbidities and on insulin, which is the same category of patients presenting higher mean annual costs, regardless of attendance to the emergency department. A cost study performed on a clinical database in the north of Italy showed that the overall direct costs for a hospitalization after severe hypoglycaemia amounted to 1161 euros [15], which is about the difference we found in the mean annual costs between patients with an ED attendance followed by hospitalization, compared to those having only an attendance to the emergency department.

We concomitantly analysed, in a very large unselected population, major determinants of severe hypoglycaemia and hyperglycaemia in DM subjects. To reach this aim, we used a zero-inflated negative binomial model, after verifying that this was the best fitting distribution for the data. This choice can complicate the interpretation because results are expressed by the combination of two effect measures, the OR of susceptibility to the event and the IRR of the event, in those who are susceptible. However, even if hypo and hyperglycaemia events are count data, they do not usually follow a Poisson distribution, and it has been highlighted that using a Poisson model may lead to biased parameter estimates and an incorrect determination of the risk factors for hypoglycaemia [27, 28, 37]. This is because the majority of patients had no event (the distribution is zero-inflated compared to Poisson) and there was more variability than expected for a Poisson distribution (i.e. the variance is greater than the mean, while in a Poisson distribution they are the same). On the contrary, zero-inflated models consider the latent group of patients never experiencing the event (no susceptible) as different from the group experiencing at least one event. The latter may be assumed following a Poisson (zero-inflated Poisson model) or negative binomial distribution (zero-inflated negative binomial model) when there is extra variability. Consequently, the predisposing factors associated with not being susceptible to ED attendances (the zero-inflated part of the model) may be different from those associated with the event rate of ED attendance for a glycaemic event, in susceptible individuals. This implies that the direction of the effect of a risk factor can be different when analysing susceptibility and event rates.

The major predictor of having any ED attendance for hypoglycaemia was type of hypoglycaemic treatment. Insulin incremented the risk by 98–99%, and the use of non-insulin anti-DM drugs at risk for hypoglycaemia, by 90%, compared to non-insulin anti-DM drugs not at risk for hypoglycaemia. For hyperglycaemia, anti-diabetic treatment influenced only the event frequency, with an eight-fold incidence rate of ED attendances for insulin users. Our study also identifies, for both acute glycaemic event types, target groups in the population needing more attention, such as the youngest and oldest patients, and patients with complicated or long-time insulin- dependent diabetes. Furthermore, we found that subjects having had a previous episode of the same type leading to ED attendance had a fivefold incidence rate of hypoglycaemia (IRR = 5.34, 95% CI 3.93–7.26) and a four-time incidence rate of hyperglycaemia (IRR = 3.68, 95% CI 1.33–10.23), which reinforces the need to identify the patients at a higher risk and organize targeted multifaceted interventions. This could include education policies for the patients and caregivers, such as improving access to specialists and implementing technologies that can continuously monitor glycaemia [38–42].

The percentage of patients hospitalized after an ED attendance for hypoglycaemia (15.3%) was lower compared to a multicentric study that included referral centres in different Italian regions (35.4%) [16]. However, the cited study referred to the period between 2010 and 2014, while our study referred to the period between 2015 and 2017. In recent years, there has been pressure, both at the regional and national levels, to reduce inappropriate hospital admission for acute diabetes complications. The Piano Nazionale Esiti (PNE National Outcome Plan) [43] showed a reduction, from 0.09 per 1000 in 2010 to 0.06 per 1000 in 2017, of the hospitalization rate for acute diabetes complications at the national level. For the area covered by the study, the same indicator ranged between 0.06 and 0.07 per 1000 for the period covered by our study. Furthermore, we included all hospital types, not only referral centres where the case mix is usually more complicated. In the same study, treatment type was not found to be a significant predictor of hospitalization after hypoglycaemia. However, the OR was 0.61 for insulin users vs. non-users. Similarly, we found that patients treated with insulin had a 40 to 50% lower rate of hospitalization compared to those treated with non-insulin anti-DM drugs not at risk for hypoglycaemia only. On the contrary, after a hyperglycaemic event, the rate of being hospitalized doubled for patients treated only with insulin and increased by almost 40% if the subject was on insulin and other anti-DM drugs.

A major strength of this study is that it is population-based and identifies the events, caused by the lack of glycaemic control, based on a validated algorithm. Furthermore, by confirming predisposing factors (e.g. young age and presence of specific comorbidities) and estimating costs related to ED attendances, our study strongly supports the fact that policies to implement glycaemic control are a priority in public healthcare, considering the risk for subsequent hospitalizations, mortality during hospitalization, and the increase in long term mortality [8, 9].

Our study only considers acute glycaemic events leading to ED attendances, which are a small fraction of all acute glycaemic events in diabetic patients. Less severe episodes may not be life-threatening and may be resolved at a small cost, but they reduce the quality of life and are related to long term complications, both for hyper and hypoglycaemia [8, 9, 44–47]. Consequently, the individual and public healthcare burden due to inadequate glycaemic control in patients with DM is underestimated. We validated the coding algorithm for acute glycaemic events in our cohort and obtained a very low false discovery rate. However, it is possible that a proportion of ED attendances, presumably the less severe ones, were generically coded (e.g. “780.7 Malaise and fatigue”), lowering the calculated incidence rate. However, as our main goal was to evaluate the factors associated with acute glycaemic ED attendances, we favoured algorithm specificity over sensitivity. A further limitation common to studies using current healthcare databases is that, while making it possible to include the entire population of an area, there is a limit to the availability of some relevant information, such as personal at-risk behaviours, body max index, or Hba1c levels. However, we constructed two proxy indicators of adequate glycaemic monitoring as a surrogate. Moreover, information on the hypoglycaemia awareness status, which is a major predictor of recurrent severe hypoglycaemic episodes, was not available in the current healthcare databases [47].

## Conclusion

The present study found that the strongest predictors of ED attendances for both hypo and hyperglycaemia in an unselected population of adults with diabetes were insulin treatment and a prior acute glycaemic event of the same type leading to ED attendance. For both events, age exerted a nonlinear effect. As they were based on routinely collected health data, our findings can be used to prospectively create an at-risk group of patients, which intensifies the need for prevention policies, such as education and glucose monitoring.

## Supplementary information

**Additional file 1.**

## Data Availability

The algorithms and sources employed to calculate variables, including ICD-9-CM and ATC codes, used in this analysis, are publicly available in the supplementary material. Individual data sharing is not possible according to sensitive data protection legislation (Italian Data Protection Authority action of 13/12/2018).

## Abbreviations

AHP: Agency for health protection
CKD: Chronic kidney disease
DM: Diabetes mellitus
DKA: Diabetic ketoacidosis
ED: Emergency department
FDR: False discovery rate
HHS: Hyperglycaemic state
IRR: Incidence rate ratio
OR: Odds ratio
PN: Peripheral neuropathy
PPV: Predictive positive value
Q1, Q3: First and third quartile
