# Supplementary material for: Emergency attendance for acute hyper- and hypoglycaemia in the adult diabetic population of the metropolitan area of Milan: quantifying the phenomenon and studying its predictors
Source: BMC Endocr Disord. 2020 May 19;20:72. doi: 10.1186/s12902-020-0546-1 (PMC7238653; doi:10.1186/s12902-020-0546-1)
Supplement: Supplementary file 1 — Additional file 1. [file 12902_2020_546_MOESM1_ESM.docx]

**Additional methods**

**ICD-9-CM codes for acute glycemic events validation**

Being the use of coding partially specific to a health system, we validated in the ED database of the Agency for Health Protection of Milan (AHP) the ICD‑9‑CM codes abstracted from a validated algorithm to identify hypoglycemia from claims^[[1]](#footnote-1)^ and those extracted from the ICD-9-CM list for hyperglycemia (Additional Table A2). The code for “Abnormal glucose tolerance of mother complicating pregnancy childbirth or puerperium” (648.8), was not included. We verified the presence of the selected diagnostic codes in the first diagnosis field of the 2015-2017 ED database of the AHP and validated the found codes in terms of false discovery rate (FDR) and positive predictive value (PPV). First, the potential ED records of acute glycemic events were identified in the ED database using all selected codes. Among them, ED events followed by hospitalization were extracted and a stratified random sample was selected^[[2]](#footnote-2)^. Strata were represented by hospital, the sampling rate was 30% of the ED followed by hospitalization for all strata and a minimum of 3 selected observations in each stratum was imposed (Surveyselect procedure, SAS software, v 9.4, SAS Institute Inc., Cary, NC, USA). Medical records of all hospital admissions of the sampled records were retrieved and four trained medical assistants, supervised by a medical epidemiologist, abstracted relevant data, including all available diagnostic codes. We compared the diagnoses of the retrieved medical record with the ICD-9-CM diagnostic code in the ED database. We calculated the FDR i.e. the proportion of cases in which each diagnostic code was used in the ED sampled records for a condition that, according to the clinical record, was not an acute glycemic event. We decided a-priori to exclude from the final algorithm all diagnostic codes with a FDR equal or greater than 30% in the sampled records. We also calculated the PPV for each type of acute glycemic event i.e. the proportion of subjects with a diagnostic code for hypoglycemia (or hyperglycemia) truly having an acute hypoglycemic (or hyperglycemic) event according to the clinical record. We a-priori set the threshold for code exclusion at a PPV lower than 80%, for either hypoglycemia or hyperglycemia, to include only codes differentiating almost certainly between hyper and hypoglycemia.

**Exclusion of ED attendances for hyperglycemia related to first DM diagnosis**

In the acute hyperglycemia analysis on the whole cohort, we wanted to exclude all ED attendances leading to the first diagnosis of DM. Consequently, in the selected random sample of the cohort used for validation, we also identified ED attendances leading to first diagnosis of DM by means of reviewing the full clinical record. We then calculated the 80th percentile of the time interval between the ED attendance leading to first diagnosis and the date of DM diagnosis included in the administrative database. This percentile was used as a cut-off to exclude ED attendances within an equal or lower time frame from the date of DM diagnosis from the acute hyperglycemia analysis.

**Additional Table A1** Summary of the Lombardy region algorithm to ascertains diabetic subjects from current health databases

| **Health database** | **ICD-9-CM codes** (at least one) | **Time interval** (years)**^a^** |
| --- | --- | --- |
| Exemption from co-payment | 013.250 | 0 -10 |
| Inpatient database  DRG field | 285 294 295 | 0 - 5 |
| I and II diagnosis fields | 250.00 250.02 250.10 250.12 250.20 250.22 250.30 250.32  250.01 250.03 250.11 250.13 250.21 250.23 250.31 250.33  250.41 250.43 250.51 250.53 250.61 250.63 250.71 250.73 250.81  250.83 250.91 2 50.93 362.01- 363.07 357.2  250.42 250.40 250.50 250.52 250.60 250.62 250.70 250.72  250.80 250.82 250.90 250.92 362.01- 363.07 357.2 | 0 -5 |
| Outpatient Drugs prescriptions | A10A*(DDD>50%)  A10B* (DDD>50%)  N03AX16 or N03AX12 (DDD>30%)  N03AX16 or N03AX12 (DDD>30%) | 0 -1 |
| Outpatients visits and diagnostic tests | 14.33 14.34 14.75 96.59.1 96.59.2 96.59.3 96.59.4 96.59.5 96.59.6 | 0 -1 |

^a^ Previous years for which the health database is searched to include the patient in the prevailing diabetic population of the index year

**Additional Table A2** ICD-9-CM Codes initially selected to detect accesses to the emergency department for acute glycemic events and those confirmed after validation on a sample of n=261 accesses through revision of the complete hospitalization clinical record

| Initially selected codes | Found codes in EA database 2015-17 | | Validation sample | | | | | | | Finally selected codes | |
| --- | --- | --- | --- | --- | --- | --- | --- | --- | --- | --- | --- |
|  | N | % | No gly. event | Hg | hg | Total | %  No gly. event | % Hg | % hg | Acute gly.  event | Specific event |
| 250.1x | 250.1x | 8.6 | 1 | 62 | 3 | 66 | 1.5 | 93.9 | 4.5 | Y | Hyper |
| 250.2x | 250.2x | 3.3 | 1 | 13 | 0 | 14 | 7.1 | 92.9 | 0.0 | Y | Hyper |
| 250.3x | 250.3x | 2.0 | 0 | 9 | 0 | 9 | 0.0 | 100.0 | 0.0 | Y | Hyper |
| 250.80 | 250.80 | 2.3 | 0 | 5 | 0 | 5 | 0.0 | 100.0 | 0.0 | Y | Hyper |
| 250.81 |  |  |  |  |  |  |  |  |  | N |  |
| 250.82 | 250.82 | 1.6 | 0 | 3 | 2 | 5 | 0.0 | 60.0 | 40.0 | Y | nd |
| 250.83 |  |  |  |  |  |  |  |  |  | N |  |
| 250.90 |  |  |  |  |  |  |  |  |  | N |  |
| 250.91 |  |  |  |  |  |  |  |  |  | N |  |
| 250.92/3 | 250.92/3 | 1.7 | 0 | 7 | 1 | 8 | 0.0 | 87.5 | 12.5 | Y | Hyper |
| 251.0 | 251.0 | 6.6 | 0 | 1 | 15 | 16 | 0.0 | 6.3 | 93.8 | Y | Hypo |
| 251.1 |  |  |  |  |  |  |  |  |  | N |  |
| 251.2 | 251.2 | 60.5 | 1 | 0 | 87 | 88 | 1.1 | 0.0 | 98.9 | Y | Hypo |
| 276.0 | 276.0 | 0.8 | 1 | 6 | 0 | 7 | 14.3 | 85.7 | 0.0 | Y | Hyper |
| 276.2 | 276.2 | 3.8 | 5 | 14 | 6 | 25 | 20.0 | 56.0 | 24.0 | Y | nd |
| 276.4 | 276.4 | 0.1 | 0 | 1 | 0 | 1 | 0.0 | 100.0 | 0.0 | Y | Hyper |
| 790.21 | 790.21 | 4.5 | 0 | 7 | 0 | 7 | 0.0 | 100.0 | 0.0 | Y | Hyper |
| 790.29 | 790.29 | 4.0 | 0 | 6 | 2 | 8 | 0.0 | 75.0 | 25.0 | Y | nd |
| 962.3 | 962.3 | 0.4 | 0 | 0 | 2 | 2 | 0.0 | 0.0 | 100.0 | Y | Hypo |
| Total |  | 100.0 | 9 | 134 | 118 | 261 | 3.4 | 51.3 | 45.2 |  |  |

Note: nd, does not discriminate between hyper and hypoglycemia

**Additional Table A3** Definition of treatment category, presence of comorbidities, and indicators of adequate glycemic monitoring

| **Factor** | **Inclusion criteria** |
| --- | --- |
| Treatment | At least one record in the outpatient drug prescription database of the following ATC codes: |
| *Insulin alone* | A10A* and no record of A10B* or A10XA​* |
| *Insulin and non-insulin anti-DM drugs* | A10A* and A10B* or A10XA*​ |
| *Anti-DM drugs including at least one at risk of hypoglycemia* | ​A10BB*, A10BX02, A10BX03, A10BD14, A10BD04,  A10BD06,  A10BD02 |
| *Other non-insulin anti-DM drugs only* | A10B* or A10XA* and no record of any of the following codes: A10BB*, A10BX02, A10BX03, A10BD14, A10BD04,  A10BD06,  A10BD02 |
| Number of comorbidities | Acromegalia and gigantism, Addison disease, Alzheimer disease, Ankylosing spondylitis, arterial and venous diseases, asthma, Basedow disease, cerebral ischemia, chronic hepatitis, hepatic cirrhosis, chronic kidney disease, chronic pancreatitis, chronic respiratory failure, congestive heart failure, COPD, Cushing syndrome, dementias, diabetes insipidus, HIV and AIDS, hyper and hypoparathyroidsm, hypertension, hypothyroidism, immune hemolytic anemia, inflammatory bowel diseases, familial and sporadic hypercholesterolemia familial, ischemic cardiomyopathy, malignant neoplasm, multiple sclerosis, myastenia gravis, optical neuromyelitis, Parkinson, Pituitary dwarfism, Psoriasis and psoriasic arthropathy, rheumatoid arthritis, Sjogren disease, systemic lupus erythematosus, systemic sclerosis, thyroiditis of Hashimoto, transplanted patients, valvular and arrhythmic cardiomyopathy (all those included in the Lombardy Region algorithms to identify patients with chronic diseases, as specified in the regional lows DR 6164 and 7655) |
| Chronic renal failure | One of the following criteria: exemption database, codes 023*,031.403,031.404; inpatient database, DRG codes 316,317 or I-II diagnosis ICD-9-CM codes V56*, 585*, 586*; outpatient drug prescription database, ATC codes V03AE02, V03AE03, V03AE01; outpatient visits and exams database, at least 70 records/year of ICD-9_CM codes 3995*‐5498* |
| Peripheral neuropathy | ICD‑9‑CM code 337.1 in the inpatient database and/or record of pregabalin (ATC code N03AX16) or duloxetine (ATC code N06AX21) in the outpatient drug prescription database |
| Glycated hemoglobin test | At least one record/year of HbA1c dosage (ICD-9-CM code 902.81) in the outpatient visit and exams database |
| Adequate number of glucose test strips for auto-monitoring, according to type of diabetes ​ | Delivery of at least the minimum number of glucose test strips according to the recommendations of the Italian Association of Diabetologist: 10 /year if taking insulin anti-DM drugs not at risk for hypoglycemia, 100/ year if taking  only anti-DM drugs including at least one at risk of hypoglycemia, 400/year if taking insulin |

**Additional Table A4** Validation sample characteristics

|  | Total | Other diagnosis | Hyperglycemia | Hypoglycemia |
| --- | --- | --- | --- | --- |
|  | N=261 | N=10 | N=134 | N=117 |
| Sex |  |  |  |  |
| Female | 130 (49.8%) | 7 (70.0%) | 74 (55.2%) | 49 (41.9%) |
| Male | 131 (50.2%) | 3 (30.0%) | 60 (44.8%) | 68 (58.1%) |
| Age class (years) |  |  |  |  |
| ≤ 44 | 28 (10.7%) | 0 ( 0.0%) | 25 (18.7%) | 3 ( 2.6%) |
| 45-54 | 20 ( 7.7%) | 0 ( 0.0%) | 15 (11.2%) | 5 ( 4.3%) |
| 55-64 | 20 ( 7.7%) | 0 ( 0.0%) | 15 (11.2%) | 5 ( 4.3%) |
| 65-74 | 41 (15.7%) | 2 (20.0%) | 24 (17.9%) | 15 (12.8%) |
| ≥ 75 | 152 (58.2%) | 8 (80.0%) | 55 (41.0%) | 89 (76.1%) |
| Deprivation index |  |  |  |  |
| I-II | 53 (20.3%) | 3 (30.0%) | 27 (20.1%) | 23 (19.7%) |
| III | 67 (25.7%) | 0 ( 0.0%) | 36 (26.9%) | 31 (26.5%) |
| IV-V | 141 (54.0%) | 7 (70.0%) | 71 (53.0%) | 63 (53.8%) |
| Type of diabetes |  |  |  |  |
| 1 | 33 (12.6%) | 0 ( 0.0%) | 28 (20.9%) | 5 ( 4.3%) |
| 2 | 228 (87.4%) | 10 (100.0%) | 106 (79.1%) | 112 (95.7%) |
| Type of diabetes |  |  |  |  |
| New diagnosis | 25 ( 9.6%) | 0 ( 0.0%) | 27 (20.2%) | 0 ( 0.0%) |
| Non-insulin dependent | 109 (41.8%) | 9 (90.0%) | 39 (29.1%) | 61 (52.1%) |
| Insulin Dependent | 127 (48.7%) | 1 (10.0%) | 68 (50.7%) | 56 (47.9%) |
| Number of comorbidities |  |  |  |  |
| None | 25 ( 9.6%) | 0 ( 0.0%) | 21 (15.7%) | 4 ( 3.4%) |
| One | 20 ( 7.7%) | 0 ( 0.0%) | 10 ( 7.5%) | 10 ( 8.5%) |
| Two or more | 216 (82.8%) | 10 (100.0%) | 103 (76.9%) | 103 (88.0%) |
| Chronic renal failure |  |  |  |  |
| No | 208 (79.7%) | 8 (80.0%) | 109 (81.3%) | 91 (77.8%) |
| Yes | 53 (20.3%) | 2 (20.0%) | 25 (18.7%) | 26 (22.2%) |
| Peripheral neuropathy |  |  |  |  |
| No | 195 (74.7%) | 9 (90.0%) | 97 (72.4%) | 89 (76.1%) |
| Yes | 66 (25.3%) | 1 (10.0%) | 37 (27.6%) | 28 (23.9%) |
| Hypoglycemia treatment^a^ |  |  |  |  |
| Insulin alone | 106 (45.3%) | 3 (30.0%) | 61 (57.0%) | 42 (35.9%) |
| Insulin and non-insulin anti-DM drugs | 29 (12.4%) | 0 ( 0.0%) | 10 ( 9.3%) | 19 (16.2%) |
| Non-insulin anti-DM drugs at risk of hypoglycemia | 46 (19.7%) | 2 (20.0%) | 11 (10.3%) | 33 (28.2%) |
| Other non-insulin anti-DM drugs only | 53 (22.6%) | 5 (50.0%) | 25 (23.4%) | 23 (19.7%) |
| Length insulin treatment |  |  |  |  |
| Less than 5 years | 57 (21.8%) | 1 (10.0%) | 31 (23.1%) | 25 (21.4%) |
| 5 years or more | 70 (26.8%) | 0 ( 0.0%) | 39 (29.1%) | 31 (26.5%) |
| No insulin treatment | 134 (51.3%) | 9 (90.0%) | 64 (47.8%) | 61 (52.1%) |
| First available glycemia (mg/dL) |  |  | 478.5 (326-607) | 38 (28.5-48) |
| Hba1c (%) |  |  | 10.4 (7.5-12.2) | 6.7 (5.9-7.3) |

^a^n=27 new diagnosis not included

1. Adit A. Ginde et al., «Validation of ICD-9-CM Coding Algorithm for Improved Identification of Hypoglycemia Visits», *BMC Endocrine Disorders* 8 (1 aprile 2008): 4, https://doi.org/10.1186/1472-6823-8-4. [↑](#footnote-ref-1)
2. Sharon L. Lohr, in *Sampling: Design and Analysis* (Cengage Learning, 2009), 95. [↑](#footnote-ref-2)
